# Non-intubated COVID-19 patients despite high levels of supplemental oxygen

**DOI:** 10.1186/s13054-021-03599-1

**Published:** 2021-05-17

**Authors:** Samuel Chosidow, Gaëtan Plantefève, Megan Fraissé, Hervé Mentec, Radj Cally, Damien Contou

**Affiliations:** Service de Réanimation Polyvalente, Centre Hospitalier Victor Dupouy, 69, Rue du Lieutenant-Colonel Prud’hon, 95100 Argenteuil, France

**Keywords:** SARS-CoV-2, COVID-19, ICU, ARDS, Mechanical ventilation, Tracheal intubation, Acute respiratory failure, ICU

Many COVID-19 patients with acute hypoxemic respiratory failure may require invasive mechanical ventilation [1]. However, deciding whether and when a patient should be intubated is complex, especially in COVID-19 patients who commonly exhibit severe hypoxemia without clinical signs of respiratory failure (the so-called silent hypoxemia) [2]. While clinical signs of respiratory failure seem to be universally acknowledged as intubation criteria [3], their precise definition is lacking. Some authors have consequently used definite thresholds to guide intubation [4], but this approach is debated [5] as an individualized strategy may be more adequate. In our ICU, only COVID-19 patients showing persistent signs of respiratory distress associated with profound hypoxemia were intubated.

We therefore aimed (1) to assess the proportion of our COVID-19 patients not receiving invasive mechanical ventilation despite high levels of supplemental oxygen (≥ 15L/min for ≥ 6 h) as well as (2) to describe their clinical and biological features on the day of worst clinical status.

We retrospectively analyzed data of COVID-19 patients (positive SARS-COV-2 RT-PCR) with acute respiratory failure admitted to our hospital between March 1st, 2020 and March 1st, 2021.

Patients were included if (1) they received 15 or more L/min of supplemental oxygen for ≥ 6 h while being hospitalized either in the wards or in ICU and if (2) they did not undergo tracheal intubation except for hypoxic cardiac arrest occurring while breathing spontaneously. Patients with a “do-not-intubate” order or still hospitalized were excluded.

Baseline was defined as the day patients met the inclusion criteria. The day of worst clinical status was defined as the day they received the highest oxygen flow with the highest respiratory rate (RR).

Among 161 patients without a “do-not-intubate” order, 49 (30%, 95% confidence interval 23–38%) did not receive invasive mechanical ventilation (Fig. 1). Baseline characteristics and description of the patients on the day of worst clinical status (number of days after hospital admission: 3 [4–6]) are detailed in Table 1. On the day of worst clinical status, the proportion of patients treated with non-invasive ventilation, high-flow nasal cannula and standard oxygen therapy was 8, 39 and 61%, respectively. The highest RR was 36 [28–40]/min while lowest SpO_2_ and PaO_2_ were 91 [90–92] % and 65 [54–73] mmHg, respectively. Fifteen patients (31%) had a RR ≥ 40/min. Median PaCO_2_ was 37 [34–42] mmHg and lactate was 1.7 [1.3–1.9] mmol/L.

**Fig. 1:**
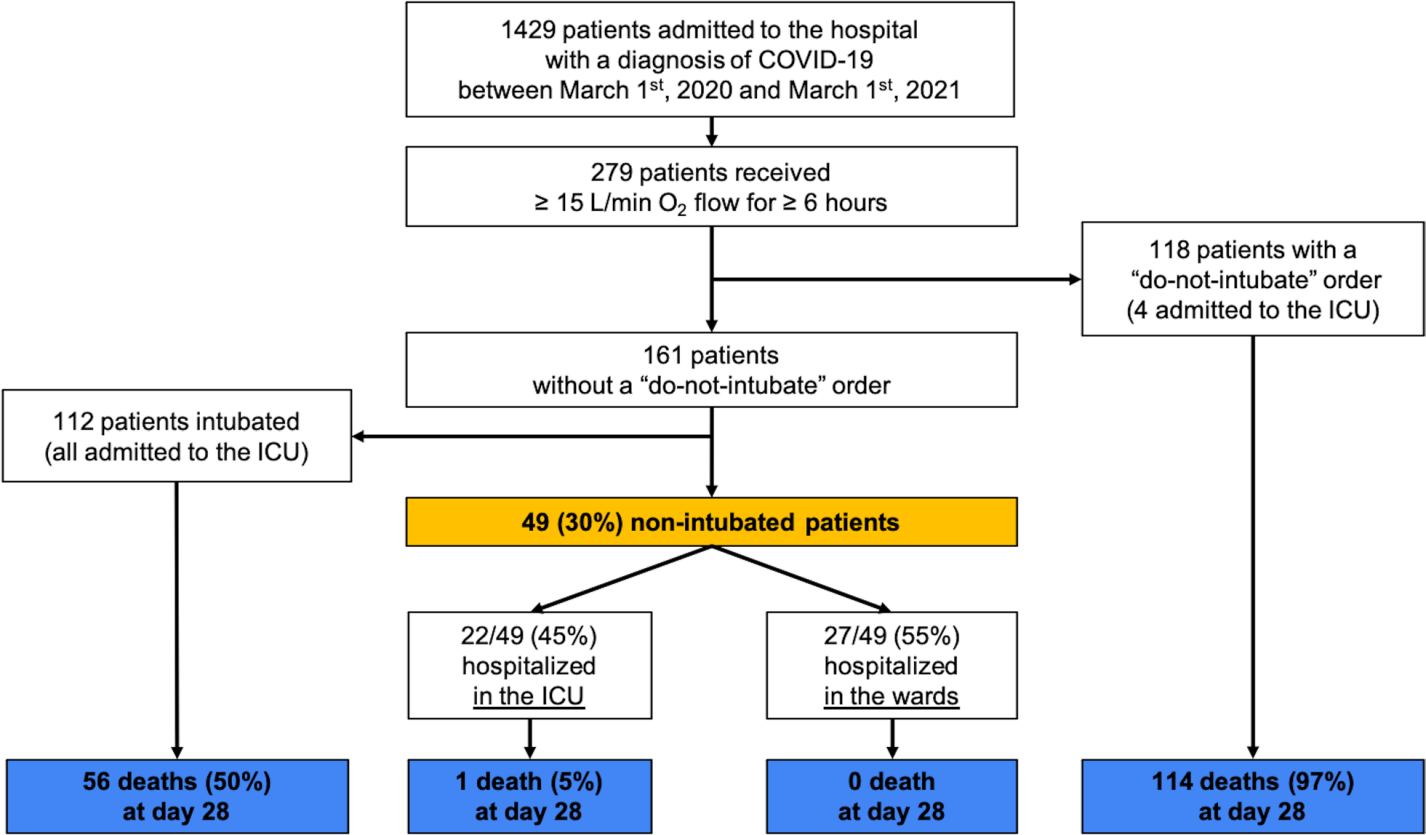
Flowchart

**Table 1:** Characteristics at baseline and on the day of worst clinical status of 49 non-intubated COVID-19 patients with acute respiratory failure hospitalized in the ICU (*n* = 22) or in the wards (*n* = 27)

	Total	Patients hospitalized in ICU	Patients hospitalized in the wards
	*n* = 49	n = 22	*n* = 27
Baseline* characteristics			
Age (years)	60 [50–66]	63 [58–67]	55 [46–63]
Male	40 (82%)	17 (78%)	23 (85%)
Diabetes	15 (31%)	9 (41%)	6 (22%)
Obesity (BMI > 30 kg/m^2^)	16 (33%)	10 (46%)	6 (22%)
Hypertension	18 (37%)	10 (46%)	8 (30%)
Chronic heart failure or coronary artery disease	3 (6%)	3 (14%)	0 (0%)
Immunosuppression	5 (10%)	3 (14%)	2 (7%)
Charlson comorbidity index	2 [1–3]	3 [1–4]	2 [0–3]
SAPS II	27 [24–30]	28 [24–30]	27 [22–30]
Days from hospital admission to baseline	3 [1–4]	2 [1–4]	3 [2–5]
Days from symptoms onset to baseline	9 [7–11]	9 [8–11]	9 [7–11]
Dexamethasone therapy	35 (71%)	16 (73%)	19 (70%)
Antibiotic therapy	30 (61%)	10 (46%)	20 (74%)
*Clinical features on the day of worst clinical status*			
Standard oxygen therapy	30 (61%)	4 (18%)	26 (96%)
Maximum oxygen flow (liters per min)	15 [15–15]	20 [15–29]	15 [15–15]
High-flow nasal cannula	19 (39%)	18 (82%)	1 (4%)
Maximum FiO_2_	1 [1–1]	1 [1–1]	1
Non-invasive ventilation	4 (8%)	4 (18%)	0 (0%)
Maximum FiO_2_	1 [1–1]	1 [1–1]	–
Highest respiratory rate (/min)	36 [28–40]	38 [35–46]	32 [28–37]
Lowest SpO_2_ (%)	91 [90–92]	90 [86–91]	91 [91–94]
Lowest mean arterial pressure (mmHg)	90 [82–98]	91 [83–100]	89 [82–96]
Highest heart rate (/min)	92 [80–104]	94 [82–108]	90 [80–98]
Highest temperature (°C)	37.7 [37–38.7]	38.0 [37–38.8]	37.5 [37–38.2]
*Biological data on the day of worst clinical status*			
Leucocytes (/mm^3^)	10,150 [8175–12975]	11,150 [9150–13875]	8850 [6600–10475]
C-reactive protein (mg/L)	119 [34–180]	149 [49–296]	115 [23–154]
pH	7.46 [7.44–7.48]	7.47 [7.43–7.48]	7.45 [7.44–7.45]
PaO_2_ (mmHg)	65 [54–73]	61 [52–69]	68 [65–102]
PaCO_2_ (mmHg)	37 [34–42]	36 [33–40]	40 [38–44]
Arterial lactate (mmol/l)	1.7 [1.3–1.9]	1.7 [1.3–2.0]	1.4 [0.9–1.7]
Creatininemia (μmol/L)	65 [57–80]	63 [56–80]	66 [57–79]
*Outcomes*			
Hypoxic cardiac arrest	1 (2%)	1 (5%)	0 (0%)
Hospital mortality	1 (2%)	1 (5%)	0 (0%)
Duration of high flow oxygen administration** (h)	82 [45–124]	114 [58–145]	72 [48–122]
Discharged home with oxygen therapy	18 (37%)	5 (23%)	13 (48%)

Continuous variables are reported as medians [quartile 1–quartile 3] and categorical variables are reported as numbers (percentages)

*Baseline was defined as the day patients met the inclusion criteria i.e. received 15 or more L/min of supplemental oxygen for ≥ 6 h

**High flow oxygen administration was defined as standard oxygen therapy with a flow of 15 L/min or more, or high-flow nasal cannula with FiO_2_ of 0.80 or more

On the day of worst clinical status, only 22 (45%) were hospitalized in ICU (Fig. 1). One of them presented hypoxic cardiac arrest while switching from high-flow nasal cannula to noninvasive ventilation.

Among the 27 (55%) patients managed in the wards, one (4%) was treated with high-flow nasal cannula while 26 (96%) were treated with standard oxygen therapy alone, with a highest oxygen flow of 15 [15–15] L/min, a highest RR of 32 [28–37]/min and a lowest SpO2 of 91 [91–94]%.

We herein report that 30% of our patients receiving ≥ 15L/min oxygen flow did not receive invasive mechanical ventilation despite significant tachypnea. Noteworthy, more than half of them were managed outside the ICU without hypoxic cardiac arrest, which could be of interest in a context of a massive inflow of critically ill COVID-19 patients.

Despite one hypoxic cardiac arrest (occurring in a patient cared for in the ICU), avoiding intubation might be feasible in some patients with high levels of supplemental oxygen and significant tachypnea. Rather than predefined SpO_2_ or RR thresholds, clinical acumen appears of paramount importance when deciding to initiate invasive mechanical ventilation.

Our results cannot be generalized to other centers nor to any other respiratory diseases than COVID-19. However, this study strengthens the idea that managing non-intubated patients with respiratory failure is a “clinical art” and that individualized patient care is necessary [6].

## Data Availability

The dataset used and analyzed for the current study is available from the corresponding author on reasonable request.

## Abbreviations

BMI: Body mass index
SAPS II: Simplified acute physiology score
ICU: Intensive care unit
